# Brazilian multicenter study on pegvisomant treatment in acromegaly

**DOI:** 10.20945/2359-3997000000159

**Published:** 2019-07-11

**Authors:** Cesar L. Boguszewski, Martha Katherine P Huayllas, Lucio Vilar, Luciana Ansaneli Naves, Antonio Ribeiro-Oliveira, Beatriz Santana Soares, Mauro Antonio Czepielewski, Julio Abucham, Silvia Regina Correa-Silva, Marcello Delano Bronstein, Raquel Soares Jallad, Felipe Gaia Duarte, Nina Rosa Musolino, Leandro Kasuki, Monica Roberto Gadelha

**Affiliations:** 1 Universidade Federal do Paraná Departamento de Medicina Interna Hospital de Clínicas Universidade Federal do Paraná Curitiba PR Brasil Departamento de Medicina Interna, Serviço de Endocrinologia e Metabologia do Paraná (SEMPR), Hospital de Clínicas, Universidade Federal do Paraná (UFPR), Curitiba, PR, Brasil; 2 Departamento de Neuroendocrinologia Hospital Brigadeiro São Paulo SP Brasil Departamento de Neuroendocrinologia, Hospital Brigadeiro, São Paulo, SP, Brasil; 3 Universidade Federal de Pernambuco Departamento de Endocrinologia Hospital de Clínicas Universidade Federal de Pernambuco Recife PE Brasil Departamento de Endocrinologia, Hospital de Clínicas, Universidade Federal de Pernambuco (UFPE), Recife, PE, Brasil; 4 Universidade de Brasília Faculdade de Ciências da Saúde Universidade de Brasília Brasília DF Brasil Faculdade de Ciências da Saúde, Universidade de Brasília (UnB), Brasília, DF, Brasil; 5 Universidade Federal de Minas Gerais Laboratório de Endocrinologia Departamento de Endocrinologia Universidade Federal de Minas Gerais Belo Horizonte MG Brasil Laboratório de Endocrinologia, Departamento de Endocrinologia, Universidade Federal de Minas Gerais (UFMG), Belo Horizonte, MG, Brasil; 6 Universidade Federal do Rio Grande do Sul Hospital de Clínicas Universidade Federal do Rio Grande do Sul Porto Alegre RS Brasil Unidade de Endocrinologia, Hospital de Clínicas, Universidade Federal do Rio Grande do Sul (UFRGS), Porto Alegre, RS, Brasil; 7 Universidade Federal de São Paulo Escola Paulista de Medicina Universidade Federal de São Paulo São Paulo SP Brasil Unidade de Neuroendocrinologia, Divisão de Endocrinologia e Metabolismo, Escola Paulista de Medicina, Universidade Federal de São Paulo (EPM-Unifesp), São Paulo, SP, Brasil; 8 Universidade de São Paulo Hospital de Clínicas Faculdade de Medicina Universidade de São Paulo São Paulo SP Brasil Unidade Neuroendócrina, Divisão de Endocrinologia e Metabolismo, Hospital de Clínicas, Faculdade de Medicina da Universidade de São Paulo (FMUSP), São Paulo, SP, Brasil; 9 Universidade de São Paulo Hospital de Clínicas Faculdade de Medicina Universidade de São Paulo São Paulo SP Brasil Divisão de Neurocirurgia Funcional, Instituto de Psiquiatria (IPq), Hospital de Clínicas, Faculdade de Medicina da Universidade de São Paulo (FMUSP), São Paulo, SP, Brasil; 10 Universidade Federal do Rio de Janeiro Centro de Pesquisa em Neuroendocrinologia Hospital Universitário Clementino Fraga Filho Universidade Federal do Rio de Janeiro Brasil Centro de Pesquisa em Neuroendocrinologia, Divisão de Endocrinologia, Hospital Universitário Clementino Fraga Filho, Universidade Federal do Rio de Janeiro (HUCFF-UFRJ);; Secretaria Estadual Da Saúde Instituto Estadual do Cérebro Paulo Niemeyer Secretaria Estadual de Saúde Rio de Janeiro RJ Brasil Divisão de Neuroendocrinologia, Instituto Estadual do Cérebro Paulo Niemeyer, Secretaria Estadual de Saúde, Rio de Janeiro, RJ, Brasil

**Keywords:** Acromegaly, human growth hormone derivatives, pegvisomant, multicenter study

## Abstract

**Objective:**

Investigate the therapeutic response of acromegaly patients to pegvisomant (PEGV) in a real-life, Brazilian multicenter study.

**Subjects and methods:**

Characteristics of acromegaly patients treated with PEGV were reviewed at diagnosis, just before and during treatment. All patients with at least two IGF-I measurements on PEGV were included. Efficacy was defined as any normal IGF-I measurement during treatment. Safety data were reviewed. Predictors of response were determined by comparing controlled versus uncontrolled patients.

**Results:**

109 patients [61 women; median age at diagnosis 34 years; 95.3% macroadenomas] from 10 Brazilian centers were studied. Previous treatment included surgery (89%), radiotherapy (34%), somatostatin receptor ligands (99%), and cabergoline (67%). Before PEGV, median levels of GH, IGF-I and IGF-I % of upper limit of normal were 4.3 µg/L, 613 ng/mL, and 209%, respectively. Pre-diabetes/diabetes was present in 48.6% and tumor remnant in 71% of patients. Initial dose was 10 mg/day in all except 4 cases, maximum dose was 30 mg/day, and median exposure time was 30.5 months. PEGV was used as monotherapy in 11% of cases. Normal IGF-I levels was obtained in 74.1% of patients. Glycemic control improved in 56.6% of patients with pre-diabetes/diabetes. Exposure time, pre-treatment GH and IGF-I levels were predictors of response. Tumor enlargement occurred in 6.5% and elevation of liver enzymes in 9.2%. PEGV was discontinued in 6 patients and 3 deaths unrelated to the drug were reported.

**Conclusions:**

In a real-life scenario, PEGV is a highly effective and safe treatment for acromegaly patients not controlled with other therapies.

## INTRODUCTION

At target cells, two distinct receptor-binding sites of the GH molecule bind to the extracellular domains of two identical pre-dimerized growth hormone receptors (GHR) forming an active 2:1 complex. Activation of GHR triggers a cascade of intracellular signaling including the JAK-STAT pathway, which is a key mediator of many genomic actions of GH (1,2). Pegvisomant (PEGV) is a genetically engineered analog of human GH with a single-amino-acid substitution at position 120 (glycine) of binding site 2 of the GH molecule that promotes GHR antagonism. Its molecular structure also comprises amino acid substitutions within GH binding site 1 and addition of polyethylene glycol moieties that increase the half-life and reduce the immunogenicity of the compound. When PEGV binds to the GHR, it inhibits IGF-I synthesis and release (3,4).

The development of PEGV has opened a new perspective in acromegaly treatment (3,4). Despite that many patients obtain biochemical control of the disease with surgery, radiotherapy and/or medical therapy with somatostatin receptor ligands (SRL) and/or cabergoline, a significant proportion of acromegaly patients remain inadequately controlled and in need of additional treatment (5). Accordingly, PEGV was approved in Europe, USA and other countries around the world for the treatment of acromegaly in patients previously treated by surgery or radiotherapy and not controlled with maximum doses of SRLs (4,6). In these cases, PEGV may be used as monotherapy or in combination therapy with SRL and/or cabergoline. In Brazil, PEGV was approved to be commercialized in 2005, but its therapeutic use is not included in the 2013 and 2018 reports on the Clinical Protocol and Therapeutic Guidelines for Acromegaly of the Ministry of Health, due to cost-effectiveness issues (7,8). As a consequence, PEGV treatment in Brazil is not reimbursed and its use is limited and only possible through “judicialization” – legal cases brought by individuals claiming their constitutional rights using the judicial system, or exceptionally, by individual out-of-pocket expenditures (9).

Sustained PEGV concentrations are crucial to its efficacy, since the drug is a reversible, competitive GHR antagonist (3). In the pivotal long-term clinical trials with PEGV, normalization of serum IGF-I was observed in up to 97% of patients treated with PEGV during 12 months with doses as high as 40 mg/day (10,11). However, efficacy has been lower in observational real-life studies, where high doses are rarely used, as the recommended dose range by the regulatory agencies is 10-30 mg daily (4). A recent report from the ACROSTUDY, an observational registry intended to collect data on PEGV therapy, including 2,090 patients treated with PEGV up to 12 years in 15 countries, found that the percentage of patients with normal IGF-I levels increased from 53% at year 1 to 73% at year 10, and not surprisingly, accompanied by an average daily dose increment from 12.8 mg at year 1 to 18.9 mg at year 10 (12). In the same report, serious adverse events related to the drug, as evaluated by the treating physicians, were described in 2.3% of the patients. Elevation of liver enzymes (ALT or AST) greater than three times in relation to pre-treatment levels was observed in 3% of the cases. Most patients (72.2%) had no change in tumor size relative to the previous scan, 16.8% had a decrease, 6.8% an increase and 4.3% had both (12).

The present real-life study was designed to obtain information on the Brazilian experience with PEGV treatment in acromegaly patients. We have collected clinical, laboratorial and radiological data from 10 reference centers specialized in the management of pituitary diseases from different regions of Brazil. The primary aim was to examine efficacy and safety of PEGV treatment, followed by identification of good predictors of therapeutic response.

## SUBJECTS AND METHODS

This is a retrospective, multicenter, real-life study, involving acromegaly patients treated with PEGV in 10 pituitary reference centers in Brazil. Diagnosis of acromegaly was made according to clinical features and elevated and/or non-glucose-suppressible serum GH levels, associated with increased IGF-I levels adjusted for age (13). A GH-secreting pituitary adenoma was evidenced by imaging at diagnosis in all cases, and the tumors were classified as micro (<1 cm) or macroadenoma (≥1 cm) according to the largest diameter.

Clinical, laboratorial and radiological characteristics of the study group were reviewed at diagnosis (DIAGN), just before PEGV treatment (PEGV-OFF) and during PEGV treatment until the last visit available (PEGV-ON). Information was obtained regarding age, gender, GH and IGF-I levels, tumor size, previous and concomitant treatments, glucose homeostasis, initial and maximal PEGV dose, and duration of treatment. All patients with at least two IGF-I measurements during PEGV treatment were included. Since this was a real-life study, serum IGF-I levels were determined at local laboratories and the results were interpreted according to the age-adjusted reference range for each specific assay used at the different settings. To standardize the results, serum IGF-I levels were expressed as a percentage of the upper limit of the normal (%ULN) reference range of each method. Indication and monitoring of PEGV treatment were carried out according to the treating physician’s clinical judgment, but data on liver enzymes, glucose and glycated hemoglobin A1c (HbA1c) levels, and tumor growth during medical treatment were determined in a routine practice in all cases. The main indication to initiate PEGV was uncontrolled disease after surgery and maximum dose of first generation SRLs and cabergoline, with or without previous radiotherapy. In few cases, PEGV was initiated in patients participating in clinical trials or due to adverse events of other medications. Efficacy was defined as any IGF-I measurement within the normal reference range adjusted for age during PEGV treatment. Safety data, including adverse events and dropouts, were also reviewed. Predictors of response were evaluated by comparing results from controlled and uncontrolled patients.

### Statistical analysis

For the descriptive analysis, categorical variables were expressed as the percentage and frequency, and the numerical variables were expressed as the median (minimum – maximum values) or mean ± SD, according to the distribution of the sample. The Mann-Whitney U test was used to evaluate the differences between hormonal levels at diagnosis, before and after PEGV therapy, as well as in the comparison between controlled and uncontrolled groups of patients. Fisher’s exact test was used to examine the association between categorical variables. The Spearman test was used for correlations. The difference was considered statistically significant when p <0.05.

## RESULTS

### Study population

A total of 109 patients (61 women and 48 men) were included in the study (Table 1). At diagnosis, the median age was 34 years (range 12-82) and a GH-secreting macroadenoma was demonstrated in 103 (95.4%) patients and a microadenoma in only 5 patients (4.7%). In one case the information about tumor size was unavailable. Median GH levels were 24.2 mg/L (0.9 – 1136 mg/L), median IGF-I levels were 1053 ng/mL (396 – 2376 mg/L) and median IGF-I ULN were 327% (120 – 756%). The results of 27 patients from one single center have been previously published (14).

**Table 1:** Clinical, laboratorial and radiological characteristics of the study population at diagnosis and just before pegvisomant treatment (PEGV-OFF)

	Total (N = 109)	Women (N = 61)	Men (N = 48)
**At diagnosis**			
Age (yrs)	34 (12 – 82)	37 (12 – 82)	34 (12 – 63)
Micro/Macroadenoma (n)	5/103	2/58	3/45
GH (µg/L)	24.2 (0.9 – 1136)	20.8 (3.0 – 680)	26.0 (0.9 – 1136)
IGF-I (ng/mL)	1053 (396 – 2376)	910 (396 – 2376)	1190 (409 – 2320)
IGF-I (%ULN)	327 (120 – 756)	313 (129 – 672)	349 (120 – 756)
**At PEGV-OFF**			
Previous surgery (%)	97 (89)	53 (87)	44 (92)
Previous radiotherapy (%)	37 (34)	21 (34)	16 (33)
Previous SRL (%)	108 (99)	60 (98)	48 (100)
Previous cabergoline (%)	73 (67)	39 (64)	34 (71)
Pre-diabetes/Diabetes (%)	53 (49)	33 (54)	20 (42)
Tumour remnant (%)	77 (71)	39 (64)	38 (79)
GH (µg/L)	4.3 (0.71 – 209)	4.0 (0.72 – 209)	4.7 (0.71 – 101)
IGF-I (ng/mL)	613 (262 – 1503)	569 (275 – 1503)	650 (262 – 1312)
IGF-I (%ULN)	209 (99 – 637)	194 (99 – 637)	220 (100 – 596)

Values are shown as median (min – max); %ULN: % upper limit of normal; SRL: somatostatin receptor ligands; no significant differences between men and women were observed.

A total of 97 (89%) patients were submitted to transsphenoidal surgery, 37 (34%) patients were treated with radiotherapy and 73 (67%) received cabergoline. All but one patient were medically treated with SRL prior to PEGV. Just before PEGV treatment (PEGV-OFF), the median GH levels were 4.3 mg/L (0.71 – 209 mg/L), median IGF-I levels were 613 ng/mL (262 – 1503 ng/mL) and median IGF-I ULN were 209% (99.7 – 637%). The only patient with an IGF-I ULN below 100% was a 15-year-old girl with an IGF-I level of 843 ng/mL (ULN = 850 ng/mL) and GH level of 2.2 mg/L after surgery, octreotide and cabergoline, who was still growing and complaining of severe joint pain. Pre-diabetes/diabetes were present in 53 (48.6%) of the patients, and 77 (71%) had a tumor remnant in the magnetic resonance imaging (MRI) (Table 1). There were no significant differences between men and women at diagnosis and at PEGV-OFF.

### Pegvisomant treatment (PEGV-ON)

The initial dose of PEGV was 10 mg/day in 105 patients. In the other 4 cases, initial dose was 15 mg, 20 mg and in two cases 40 mg/week in two applications (5.7 mg/day). In 12 (11%) of the cases, PEGV was used as monotherapy in the whole period of observation, while it was associated with SRL in 92 and/or with cabergoline in 23 patients, at different periods of time throughout the treatment, following treating physician’s judgement. The median duration of PEGV exposure was 30.5 months (2 – 124 months), with a median maximum dose of 10 mg/day. The initial dose was maintained during the entire period of PEGV exposure in 58 (53%) patients, it was increased to 15 mg/day in 12 (11%), to 20 mg/day in 26 (24%) and to 30 mg/day in only 11 (10.2%). None of the patients received daily dose of PEGV higher than 30 mg/day (Table 2). No statistically significant differences between men and women were noted at PEGV-ON.

**Table 2:** Therapeutic approach and efficacy of pegvisomant (PEGV) treatment in Brazilian acromegaly patients

	Total (N = 109)	Women (N = 61)	Men (N = 48)
PEGV initial dose 10 mg/day (%)	105 (96)	58 (95)	47 (98)
PEGV maximum dose (mg/day) 5,7 (%) 10 (%) 15 (%) 20 (%) 30 (%)	2 (1.8) 58 (53) 12 (11) 26 (24) 11 (10.2)	2 (3.3) 36 (59) 5 (8.2) 13 (21.3) 5 (8.2)	0 (0) 22 (45.8) 7 (14.6) 13 (27.1) 6 (12.5)
PEGV monotherapy (%)	12 (11)	8 (13.1)	4 (8.3)
PEGV + SRL (%)	92 (84.4)	53 (87)	39 (81.2)
PEGV + cabergoline (%)	23 (21)	11 (18)	12 (25)
PEGV exposure (months)	30.5 (2 – 124)	30.0 (2 – 110)	30.5 (2 – 124)
IGF-I (ng/mL)	206 (30 – 946)	192 (30 – 828)	220 (88 – 946)
IGF-I (%ULN)	84.4 (12.6 – 253.3)	72.5 (12.6 – 253.3)	89.5 (21.6 – 246)
Normal IGF-I on PEGV (%)	80 (74.1)	44 (73.3)	36 (75.0)

Values are shown as median (min – max); %ULN, % upper limit of normal; SRL, somatostatin receptor ligands; no significant differences between men and women were observed.

**Figure 1:**
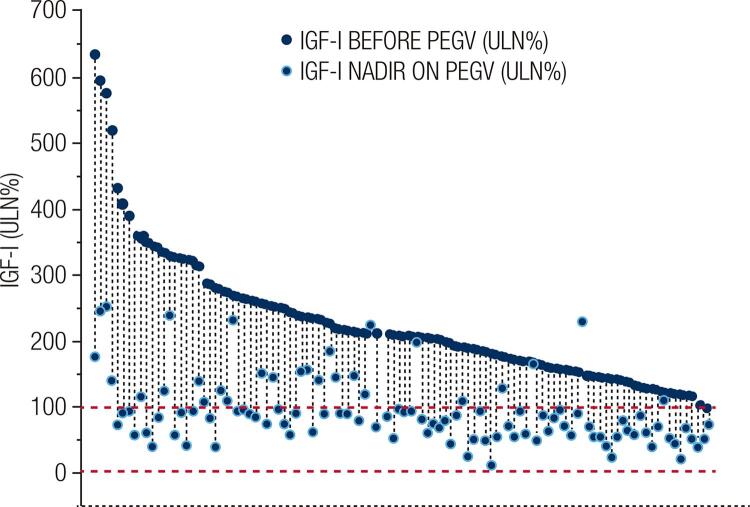
Individual IGF-I levels, expressed as a percentage of the age-adjusted upper limit of normal (ULN) range, before (dark blue circles) and after (light blue circles) pegvisomant (PEGV) treatment in 109 Brazilian patients with acromegaly.

**Figure 2:**
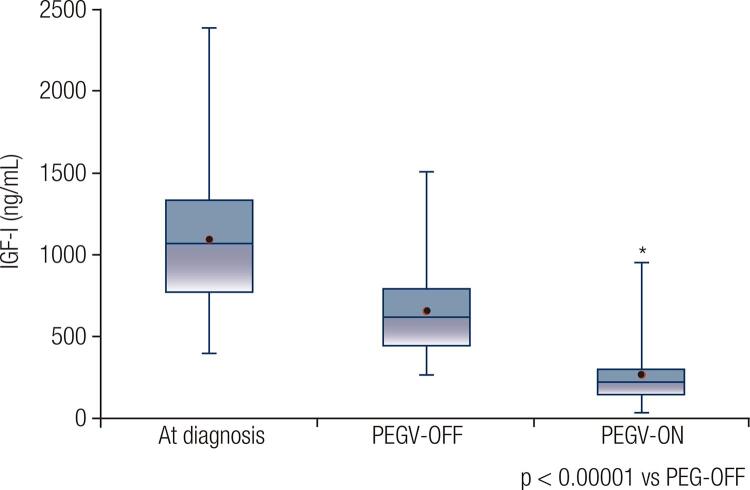
IGF-I levels at diagnosis, just before pegvisomant treatment (PEGV-OFF) and nadir values after pegvisomant treatment (PEGV-ON) in 109 Brazilian patients with acromegaly. Horizontal lines in the box plots represent from top to bottom: 97, 75, median, 25, and 3 percentiles, and the dots represent mean values.

Normalization of serum IGF-I levels at any point during therapy was obtained in 80 (74.1%) patients: 11 (92%) in monotherapy and 69 (71%) in combined treatment. The median maximum dose of PEGV in monotherapy was 15 mg/day and in combined treatment was 10 mg/day, but these values were not statistically different. Figure 1 shows individual IGF-I ULN values before and after PEGV treatment in the whole cohort. In the PEGV-ON period of the study, median IGF-I levels and IGF-I ULN were 206 ng/mL (30 – 946) and 84.4% (12.6 – 253.3), respectively, values significantly lower in relation to PEGV-OFF values (p < 0.00001; Figure 2). There was no difference in the PEGV treatment outcomes comparing patients who were previously irradiated with those who were not. Normal IGF-I was observed in 26 of 37 patients (70.3%) previously treated with radiotherapy and in 54 of 72 patients (75%) not irradiated. Improvement of glycemic control determined by fasting glucose and HbA1c levels was documented in 30 of 53 patients (56.6%) with pre-diabetes/diabetes at PEGV-OFF period.

Tumor enlargement was described in 5 (6.5%) of 77 patients with tumor remnant at PEGV-OFF. Only one of the 5 patients was in monotherapy, and in all cases it was observed in aggressive tumors and considered unrelated to PEGV treatment by the treating physician. Elevation of liver enzymes greater than 3 times in relation to pre-treatment levels occurred in 10 (9.2%) patients, lipohypertrophy, pain at injection site and headache were reported by 5, 3 and 2 patients, respectively (Table 3). Six women and no man discontinued PEGV treatment, and the causes were lipohypertrophy, pain at injection site, headache, elevation of liver enzymes, allergic reaction, and pregnancy. Three deaths were observed, all considered not related to PEGV therapy: a sudden death of unknown cause, one due to stroke and one caused by severe heart failure.

**Table 3:** Safety data on pegvisomant treatment in Brazilian acromegaly patients

	Total (N = 109)	Women (N = 61)	Men (N = 48)
Tumour enlargement (%)*	5 (6.5)	5 (12.8)	0 (0)
Elevation of liver enzymes (%)	10 (9.2)	5 (8.2)	5 (10.4)
Lipohypertrophy (%)	5 (4.6)	4 (6.5)	1 (2.1)
Pain at injection site (%)	3 (2.7)	3 (4.9)	0 (0)
Headache (%)	2 (1.8)	2 (3.3)	0 (0)
Treatment discontinuation (%)^#^	6 (5.5)	6 (9.8)	0 (0)
Death (unrelated to therapy) (%)^§^	3 (2.7)	2 (3.3)	1 (2.1)

* In 77 patients with tumor remnant at beginning of treatment (39 women, 38 men).

^#^ Lipohypertrophy, pain at injection site, headache, elevation of liver enzymes, allergic reaction, pregnancy.

^§^ sudden death (unknown cause), stroke, severe heart failure.

### Predictors of treatment response

Table 4 shows the comparison of clinical and laboratorial characteristics at diagnosis and PEGV-OFF phases between patients controlled and uncontrolled at PEGV-ON. The median duration of PEG exposure was 41 (2 – 120) months in the controlled group, which was significantly higher than 22 (3 – 124) months observed in the uncontrolled group (p = 0.03). At PEG-OFF, median GH levels were significantly lower in the controlled patients [3.75 mg/L (0.71 – 101) *vs* 7.8 mg/L (1.03 – 209), p < 0.01)], as well as median IGF-I levels [570 ng/mL (262 – 1155) *vs* 717 ng/mL (279 – 1503), p < 0.001)] and IGF-I ULN [194% (99 – 434) *vs* 241% (124 – 637), p < 0.001)]. There was no difference between the two groups regarding age at diagnosis, gender, previous radiotherapy, and the presence of tumor remnant or pre-diabetes/diabetes.

**Table 4:** Comparison of clinical, laboratorial and radiological features between controlled (normal IGF-I levels on pegvisomant (PEGV-ON) and uncontrolled acromegaly patients

	Controlled (N = 80)	Not controlled (N = 29)	P-value
Age at diagnosis (yrs)	35 (12 – 75)	31 (12 – 82)	0.09
Female (%)	44 (55)	17 (58.6)	0.73
Tumour Remnant (%)	55 (68.7)	22 (78.6)	0.32
Previous radiotherapy (%)	26 (32.5)	11 (37.9)	0.59
Pre-Diabetes/Diabetes (%)	36 (45)	17 (58.6)	0.20
PEGV exposure (months)	41 (2 – 120)	22 (3 – 124)	0.03
GH PEGV-OFF (µg/L)	3.75 (0.71 – 101)	7.80 (1.03 – 209)	0.01
IGF-I PEGV-OFF (ng/mL)	570 (262 – 1155)	717 (279 – 1503)	< 0.001
IGF-I PEGV-OFF (ULN%)	194 (99 – 434)	241 (124 – 637)	< 0.001

PEGV-OFF: just before PEGV treatment; %ULN: % upper limit of normal.

## DISCUSSION

This first Brazilian multicenter real-life study has confirmed the conclusion of previous clinical trials and observational studies that PEGV is an effective and safe medical therapy to acromegaly patients. In fact, PEGV might be the only therapeutic option to obtain biochemical control in a subgroup of patients who remain with active disease after surgery, radiotherapy, SRLs and cabergoline (15). Although the analysis of surveillance data suggests a biochemical control rate in approximately 75% of patients treated with PEGV as first-line monotherapy, PEGV is preferably a second-line medical therapy, as it has no effect on the GH-secreting adenoma (13,15,16). This recommendation is in line with the Brazilian experience, as in our cohort 89% of patients were operated, all but one patient was treated with SRL, 67% received cabergoline and roughly one-third was treated with radiotherapy before PEGV.

Acromegaly is associated with enhanced mortality and a high morbidity when normalization of GH and/or IGF-I levels is not achieved (17). There is some evidence that total direct treatment costs are higher for patients with uncontrolled compared to those with controlled disease (18). Thus, one should expect that a medication that promotes biochemical control rate in two-third of patients, as observed in our study, would result in a cost-effective treatment. However, our study was not designed to evaluate this outcome and there are still many unanswered questions about the benefits of controlled versus uncontrolled disease in relation to the economic impacts to health systems (8,18).

The SRLs octreotide and lanreotide are the first-line medical therapy in most acromegaly patients, with biochemical control observed in 20-40% of patients, depending on the study design (13,15,19-22). The new generation SRL pasireotide is effective in up to 20% of patients who do not respond to octreotide or lanreotide (23,24). Our result of 74.1% of patients achieving normal IGF-I confirms that PEGV is one of the most effective drugs to obtain biochemical control in acromegaly. This percentage is slightly lower than that observed in a previous Brazilian single center study, in which 85% of 27 patients were successfully treated (14). In both studies the median daily dose of PEGV was 10 mg, and no patient received doses higher than 30 mg after a median time of exposure of 30.5 months. The results from the single center and the present multicenter study in Brazil are surprisingly better than expected, as in the last report from the ACROSTUDY, an average dose of 12.8 mg was associated with normal IGF-I in 53% of the patients at year 1 (12). A percentage of 73% of normal IGF-I, similar to that of our study, was described in the ACROSTUDY only at year 10 with a higher average dose of 18.9 mg (12). Besides the differences attributable to variable doses of PEGV in the studies, several other factors have been associated with the therapeutic responses to PEGV. Age, gender, body mass index, previous radiotherapy and the presence of diabetes have been suggested as predictive of the PEGV dose required for normalization of IGF-I levels (25-27). Height and weight were not available in our study, while age, gender, previous radiotherapy and diabetes were not correlated to PEGV dose and were not predictive of response to PEGV. On the other hand, we found that pre-treatment GH and IGF-I levels and time of exposure were predictors of response. In agreement, baseline GH and IGF-I levels have been correlated with the PEGV dose required to normalize serum IGF-I in patients with active acromegaly (28).

Another possible factor influencing the results of PEGV therapy in acromegaly among different studies is the number of patients in monotherapy and in combined therapy with SRLs and/or cabergoline. PEGV has shown efficacy rates as high as 97% when given in combination with an SRL and delivered once or twice weekly (29). In addition, effectiveness might continue after SRL discontinuation (30). This number is higher than that reported on long-term treatment with PEGV as monotherapy in the ACROSTUDY, where normal IGF-I was observed in 67.5% of patients at 5 years of therapy (31). In our study, PEGV was used as monotherapy in the whole period of observation in only 11% of the patients, while the vast majority of patients were also exposed to SRL at variable times during follow-up, and a smaller proportion was co-exposed to cabergoline. These percentages are fairly similar to those of the Brazilian single center study (14), and might explain the good efficacy results observed in both real-life Brazilian studies, even that a large proportion of patients were treated with the lowest doses of PEGV. Accordingly, it has been previously demonstrated that the combined therapy of SRLs with PEGV can normalize IGF-I levels in virtually all patients and control tumor size in a vast majority of patients if an adequate dose of PEGV is used (32).

In our study, approximately 60% of patients treated with PEGV presented an improvement of their pre-diabetes/diabetes status, which is very relevant in comparison to other medications that are neutral or might even worsen glycemic control. A recent meta-analysis of prospective interventional studies have shown that PEGV, in monotherapy or combined with SRLs, improves glucose metabolism by reducing fasting plasma glucose and insulin levels, HbA1c, and insulin resistance, independently of disease control (33). In agreement with that, a consensus statement on acromegaly therapeutic outcomes has recommended PEGV as the best medical option for patients with impaired glucose tolerance unresponsive to first-generation of SRLs, due to its beneficial effect on insulin sensitivity and glucose tolerance (15).

The safety profile in our experience was fairly similar to what has been described in previous studies (4,6). One of the major concerns related to PEGV therapy is the potential growth of the somatotroph pituitary adenoma due to reduced feedback mechanisms. Tumor enlargement was described in 3.2% out of 936 patients with a minimum of two available MRI examinations in a comprehensive review of 1288 subjects in ACROSTUDY (34). In the most recent ACROSTUDY report, 6.8% of 2090 patients had an increase in tumor size, very similar to our findings (12). In all of our cases, tumor growth was not considered as a consequence of PEGV treatment and occurred in patients harboring aggressive GH-secreting adenomas unresponsive to multimodal therapies. It is possible that SRLs withdrawal might contribute to tumor growth in some cases, but it was not possible to evaluate the influence of SRLs withdrawal in our study due to its real-life design. Mild elevation of liver enzymes occurs in 9.2% of our cohort, as compared to 2.5 % described in other studies (4), and it was a reason for PEGV discontinuation in one female patient. In the other cases, liver enzymes returned to normal upon temporary drug discontinuation, dose reduction or without intervention. There were other five dropouts, all in women, due to lipohypertrophy, pain, headache, allergic reaction, and pregnancy. There were also three deaths during follow-up that were considered unrelated to PEGV therapy by the treating physicians.

In summary, PEGV treatment has been proved to be highly effective and safe in a large cohort of Brazilian patients with active acromegaly and not controlled with all other available therapeutic modalities. It might be particularly useful in patients with impaired glucose tolerance. In our study, lower pre-treatment GH and IGF-I levels and longer time of exposure were associated with better response to PEGV therapy.
